# Interval Progress in Gender Diversity Among Obstetrics and Gynecology Leadership Positions

**DOI:** 10.1097/og9.0000000000000158

**Published:** 2026-03-05

**Authors:** Minhazur Sarker, Cynthia Gyamfi-Bannerman, Ann M. Bruno

**Affiliations:** Department of Obstetrics, Gynecology and Reproductive Science, Division of Maternal Fetal Medicine, University of California San Diego, La Jolla, California; and Department of Obstetrics and Gynecology, Division of Maternal Fetal Medicine, University of Utah Health, Salt Lake City, Utah.

## Abstract

The proportion of women in obstetrics and gynecology leadership positions has increased significantly over the past decade but remains below workforce representation.

Leadership in obstetrics and gynecology has historically been male dominated despite an increasingly female workforce. Representative gender diversity among leadership positions in numerous surgical subspecialties, including obstetrics and gynecology, has been well documented.^1–5^ In 2012, an editorial published in *Obstetrics & Gynecology* highlighted the growing proportion of women physicians choosing obstetrics and gynecology such that “within 10 years, our members and our leaders will be mostly women.”^6^ Shortly afterward, a study of department chairs, editors, and professional society leaders in obstetrics and gynecology found that only 21.1% of leadership positions were held by women.^4^ The interval proportion of women in obstetrics and gynecology leadership positions has not been reported, but it was hypothesized that the prediction made in 2012 may be realized over time.^4,7^ We aimed to evaluate the proportion of leadership positions in obstetrics and gynecology held by women 10 years later.

## METHODS

This cross-sectional analysis was conducted in June 2025 of obstetrics and gynecology leadership positions across three categories: 1) department chairs associated with the Council of University Chairs of Obstetrics and Gynecology (list obtained on June 4, 2025),^8^ 2) editor-in-chief and deputy editors among the top 20 obstetrics and gynecology journals by impact factor (obtained from SCImago Journal & Country Rank online database on June 5, 2025),^9^ and 3) presidents of obstetrics and gynecology professional societies (Appendix 1, available online at http://links.lww.com/AOG/E564). The methodology mirrors that of the 2012 study.^4^ Publicly available data were searched to identify individuals holding each leadership position. The proportion of these leaders identifying as women was determined according to reported gender on institutional profiles or preferred pronouns. We compared the current proportion of women in obstetrics and gynecology leadership with that from 2012^4^ and with the expected proportion defined by the number of women physicians choosing obstetrics and gynecology from the 2022–2025 National Resident Matching Program results.^10^ Secondary analysis evaluated the proportion of women by obstetrics and gynecology leadership category. We used STATA IC 15.1 for χ^2^ and Fisher exact test analyses as appropriate. This study was exempt from IRB oversight.

## RESULTS

**Fig. 1.**
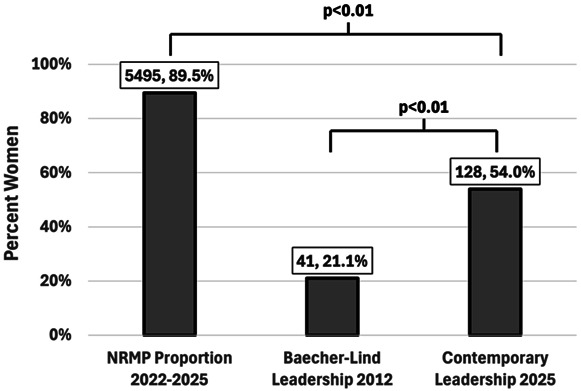
Proportion of women in obstetrics and gynecology leadership positions. Contemporary leadership reflects the proportion from this analysis relative to the 2012 analysis by Baecher-Lind^4^ and the proportion of women physicians choosing obstetrics and gynecology as per National Residency Matching Program (NRMP).

**Fig. 2.**
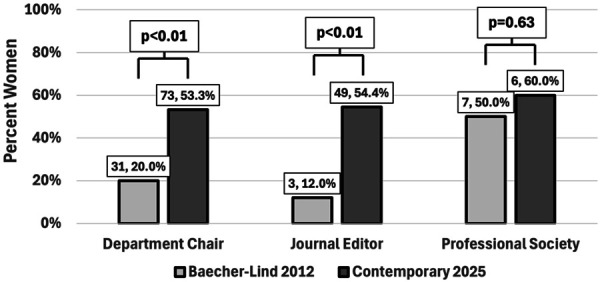
Proportion of women in obstetrics and gynecology leadership positions by position type. Representation of specific leadership position (department chair, journal editor, or president of professional society) relative to 2012 analysis by Baecher-Lind.^4^ Professional societies included AAGL, American Board of Obstetrics and Gynecology, Inc, American College of Obstetricians and Gynecologists, Association of Professors of Gynecology and Obstetrics, American Society for Reproductive Medicine, American Urogynecologic Society, Society of Family Planning, Society of Gynecologic Oncology, Society for Maternal-Fetal Medicine, and Society for Academic Specialists in General Obstetrics and Gynecology.

Of 237 obstetrics and gynecology leadership positions identified, department chairs, editorial leadership, and society presidents made up 137 (57.8%), 90 (38.0%), and 10 (4.2%) positions, respectively. Women held 128 of the 237 leadership positions (54.0%) identified. Although the proportion is significantly higher in 2025 compared with 2012 (54.0% vs 21.1%, *P*<.01), the proportion remains less than the proportion of women physicians entering obstetrics and gynecology from 2022 to 2025 (54.0% vs 89.5%, *P*<.01, Fig. 1). When stratified by category of leadership position and compared with 2012, a significantly higher proportion of women serve as department chairs (20.0% vs 53.3%, *P*<.01) and editorial leadership (12.0% vs 54.4%, *P*<.01) but not as professional society presidents (50.0% vs 60.0%, *P*=.63, Fig. 2).

*Sarker. Gender Diversity in Obstetrics and Gynecology Leadership. O&G Open 2026*.

*Sarker. Gender Diversity in Obstetrics and Gynecology Leadership. O&G Open 2026*.

## DISCUSSION

We found the proportion of women serving in obstetrics and gynecology leadership positions in 2025 to be higher than previously published in 2012. With over 50% of leadership positions in each category held by women, the prediction that “our members and our leaders will be mostly women” has been met.^4,6^ However, as the number of women entering into obstetrics and gynecology residency has increased over time, the current proportion of women in leadership is not representative of physicians pursuing careers in obstetrics and gynecology in the past few years (2022–2025). The previously set goal (more than 50%), therefore, is a low bar for the current proportion of women entering the field. Additional attention and effort are likely needed to bring the proportion of women in leadership into alignment with the proportion of women entering careers in obstetrics and gynecology.

Prior studies have found consistent and persistent nonrepresentative gender diversity among leadership positions in surgical subspecialties, including cardiothoracic, orthopedic, plastic, and neurosurgery.^1–3^ In a systematic review of 94 studies on institutional leadership, Levy et al^3^ found that academic medicine, especially among surgical subspecialties, lacked gender diversity. Among the five obstetrics and gynecology studies included, only one evaluated department chair positions and reported that 28.7% were held by women.^5^ Thus, even female-dominated specialties such as obstetrics and gynecology lack gender diversity among leadership positions. Our findings are also similar to prior work evaluating obstetrics and gynecology graduate medical education leadership positions such as residency or fellowship directors.^5^

This study has limitations. First, we used online reported gender or pronouns to ascertain gender, which may not always be accurate. The analysis was limited to chairs affiliated with Council of University Chairs of Obstetrics and Gynecology and thus may exclude some community hospitals or private practice settings. Finally, our evaluation of professional societies was not comprehensive of all societies.

This study also has strengths. The used of self-identified gender as opposed to biologic sex is a better representation of gender diversity. The data accessed from the National Resident Matching Program and Council of University Chairs of Obstetrics and Gynecology are publicly available sources of reliable and reproducible data.

The increased proportion of women serving in obstetrics and gynecology leadership positions in this contemporary evaluation supports progress over time, but ongoing attention and work are needed for the proportion to reflect the current gender representation of trainees entering the field.
